# Polymorphisms of the genes *ABCG2*, *SLC22A12 *and *XDH *and their relation with hyperuricemia and hypercholesterolemia in Mexican young adults

**DOI:** 10.12688/f1000research.46399.2

**Published:** 2021-09-24

**Authors:** Juan Manuel Vargas-Morales, Martha Guevara-Cruz, Celia Aradillas-García, Lilia G. Noriega, Armando Tovar, Jorge Alejandro Alegría-Torres

**Affiliations:** 1 Laboratorio de Análisis Clínicos, Facultad de Química, Universidad Autónoma de San Luis Potosí, San Luis Potosí, San Luis Potosí, 78210, Mexico; 2 Departamento de Fisiología de la Nutrición, Instituto Nacional de Ciencias Médicas y Nutrición Salvador Zubirán, Ciudad de México, México, 14080, Mexico; 3 Centro de Investigación Aplicada en Ambiente y Salud, CIACYT-Facultad de Medicina, Universidad Autónoma de San Luis Potosí, San Luis Potosí, San Luis Potosí, 78210, Mexico; 4 Departamento de Farmacia, Universidad de Guanajuato, Guanajuato, Guanajuato, 36050, Mexico

**Keywords:** ABCG2, SLC22A12, XDH, hyperuricemia, hypercholesterolemia

## Abstract

**Background:** Hyperuricemia is a pathological condition associated with risk factors of cardiovascular disease. In this study, three genetic polymorphisms were genotyped as predisposing factors of hyperuricemia.

**Methods:** A total of 860 Mexicans (129 cases and 731 controls) between 18 and 25 years of age were genotyped for the *ABCG2* (Q191K), *SLC22A12* (517G>A), and *XDH *(518T>C) polymorphisms, as predisposing factors of hyperuricemia. Biochemical parameters were measured by spectrophotometry, while genetic polymorphisms were analyzed by real-time PCR. An analysis of the risk of hyperuricemia in relation to the variables studied was carried out using a logistic regression.

**Results:** Male sex, being overweight or obese, having hypercholesterolemia or having hypertriglyceridemia were factors associated with hyperuricemia ( *p* ≤ 0.05). The *ABCG2* polymorphism was associated with hyperuricemia (OR = 2.43, 95% CI: 1.41-4.17, *p* = 0.001) and hypercholesterolemia (OR = 4.89, 95% CI: 1.54-15.48, *p* = 0.003), employing a dominant model, but only in male participants.

**Conclusions**: The *ABCG2* (Q191K) polymorphism increases the risk of hyperuricemia and hypercholesterolemia in young Mexican males.

## Introduction

Hyperuricemia is an abnormal metabolic trait defined as serum uric acid levels above 6 and 7 mg/dL for women and men, respectively ( Bardin & Richette, 2014), and is associated with other cardiometabolic risk factors such as obesity, diabetes, hypertension, and dyslipidemia ( Martínez-Quintana *et al.*, 2016; Zuo *et al.*, 2016). Similarly, hyperuricemia can be a predictor of impaired kidney functions as well as kidney disease progression ( Galán *et al.*, 2018; Hsu *et al.*, 2009, and Pérez-Navarro *et al*., 2020). Serum uric acid levels depend on many factors including diet, sex, lifestyle, and alcohol consumption, as well as genetic heredity. In fact, some genes involved in the metabolism of purines or urates have versions that appear to predispose to a hyperuricemic condition. Some of these are the *ABCG2* gene that encodes a membrane transporter, which exports urates to the kidney and intestine ( Woodward *et al.*, 2009); and the *SLC2A9* gene that encodes a kidney protein called GLUcose Transporter 9 (GLUT9), an important flow regulator of urates in the proximal tubules ( Caulfield *et al.*, 2008). In the same way, the *XDH* gene gives rise to an enzyme denominated xanthine dehydrogenase, which breaks down purines from nucleic acids, specifically the hypoxanthine‒xanthine‒urate conversion pathway ( Harrison, 2002).

Recent studies have shown that genetic polymorphisms in *ABCG2* and *SLC2A9* genes are prevalent in the Mexican population and may contribute to an abnormal condition of hyperuricemia, even at young ages ( Macías-Kauffer *et al.*, 2019; Rivera-Paredez *et al.*, 2019). Interestingly, there appears to be no reports to date in the literature on *XDH* gene polymorphisms in the Mexican population. Therefore, the aim of this study was to genotype the polymorphisms of the genes *ABCG2* (Q191K), *SLC22A12* (517G>A), and *XDH* (518T>C) in 860 young Mexican volunteers aged between 18 and 25 years of age as predisposing factors of hyperuricemia associated with risk factors of cardiovascular disease.

## Methods

### Study design and ethical statement

A cross-sectional design and a convenience sample of 860 subjects was coducted in the City of San Luis Potosí, México. All participants were college applicants during the spring period of 2017. Data collection was carried out according to the recruitment protocols of the University of San Luis Potosí, since applicants receive a clinical evaluation as part of the admission process. This project was approved by two Bioethics Committees: the former at the National Institute of Medical Sciences and Nutrition Salvador Zubirán (Approval # FNU-669-13-15-2), and the latter at the Faculty of Chemistry of the University of San Luis Potosí (Approval # CEID2017105-S). All participants provided written informed consent.

### Participants

A total of 860 applicants to the state University of San Luis Potosí, Mexico in 2017, were included in this study (448 men and 412 women). All participants were approached to take part in the study by phone and email by means of an open invitation explaining the goals of the study. Even though the invitation was open, only some accepted to participate. Participants were not paid to take part. All individuals who provided written informed consent and met the inclusion criteria were included. The inclusion criteria which were as follows: aged between 18–25 years; born in the Mexican state of San Luis Potosí; and provided written informed consent.

### Clinical assessment and sample collection

Since all participants at the University of San Luis Potosí, Mexico receive a clinical evaluation as part of the admission process, we accessed the university medical records. The weight in kilograms and height in meters of each participant was obtained for anthropometry using a digital scale (UM-081 model; Tanita, Tokyo, Japan) and a stadiometer (Seca 213, 2009; Seca, Hanover, MD, USA), respectively. From this data, the Body Mass Index (BMI) was calculated (kg/m ^2^). Systolic and diastolic blood pressure were taken as the mean of two readings at a 5-min interval after 5 min in a seated position, employing the Omrom model HBP-1300 portable meter (Omron Healthcare, Inc., IL, USA). Also, 6 mL of blood was obtained by venipuncture after a 12-h fast; serum was obtained by centrifuging at 1,000 x g for 10 min using a laboratory centrifuge Z306 Benchmark Scientific (NY, USA), and processed inmmediately. An addition, 3 mL of blood was collected and stored in ethylenediaminetetraacetic acid (EDTA) tubes (Vacutainer®) for subsequent DNA purification. All samples were maintained at -20°C until their analysis.

### Measurement of biochemical profiles

Serum was used to measure uric acid, glucose, total cholesterol, low-density lipoprotein cholesterol (LDL-C), high-density lipoprotein cholesterol (HDL-C), and triglycerides by spectrophotometry utilizing chemistry reagents (Paramedical S.r.l., Italy), specially developed to work with Mindray BS 300 Auto Chemistry Analyzer (Mindray, Shenzhen, China), with the following catalog numbers: uric acid (PDIBS200040), glucose (PDIBS200020), total cholesterol (PDIBS200030), LDL-C (PDIBS200170) HDL-C (PDIBS200160), and triglycerides (PDIBS200060).

### Genotyping of polymorphisms

DNA was isolated from whole blood using the QIAamp DNA Blood Mini Kit (QIAGEN, Hilden, Germany). The quality of DNA samples was verified by spectrophotometry using NanoDrop™ 2000. Polymorphisms of the genes *ABCG2* (rs2231142), *SLC22A12* (rs476037), and *XDH* (rs1042039) were measured by allelic discrimination real-time polymerase chain reaction (PCR) with the Taqman probe (Applied Biosystems®).

All PCR reactions were carried out using an ABI Prism 7900 HT detection system in 96-well plates (Applied Biosystems®). Reactions were adjusted to a final volume of 10 µL including Master mix probes and 50 ng/µL of genomic DNA, with the following amplification protocol: denaturation 94° for 9 min; followed by 50 cycles of denaturation at 95°C for 30 s, and annealing and extension at 68°C for 11 min. Negative controls and duplicated samples were included to check the accuracy of the genotyping.

### Statistical analysis

After analyzing the data distribution, comparison of medians between groups (normal uricemia vs. hyperuricemia) was carried out with the Mann‒Whitney *U* test for continuous variables. Allelic and genotypic frequencies as well as the Hardy‒Weinberg equilibrium were calculated for each polymorphism utilizing the chi-square test. Likewise, a logistic regression analysis was used to calculate the risk of hyperuricemia in relation to the variables studied and the influence of the different genotypes on uric acid levels in blood through a dominant model. The level of significance was set at *p* <0.05. Data were analyzed using SPSS version 19.0 statistical software.

## Results

In total, 860 participants were included in this study: 52% men and 48% women ( Alegría-Torres, 2021). Participant characteristics, as well as the allelic and genotypic frequencies of the *ABCG2* (Q191K), *SLC22A12* (517G>A) and *XDH* (518T>C) gene polymorphisms, are presented in Table 1. Medians and genotypic distribution between hyperuricemic and normouricemic participants were analyzed using the Mann-Whitney *U* test, finding differences for sex, BMI, systolic and diastolic pressure, triglycerides, total cholesterol, and LDL-C ( *p* = <0.001 for all), as well as a marginal distribution difference for the *ABCG2* (Q191K) polymorphism corresponding to G/T alleles ( *p* = 0.056), therefore other genetic models were tested later. All the genotypes analyzed were in Hardy‒Weinberg equilibrium: *ABCG2* (Q191K) *X* ^2^ = 1.82X10 ^-6^, *p* = 0.9; *SLC22A12* (517G>A) *X* ^2^ = 0.03, *p* = 0.84; and *XDH* (518T>C) *X* ^2^ = 0.08, *p* = 0.76. Both allelic and genotypic distributions of the three polymorphisms studied did not differ significantly between men and women ( Table 2).

**Table 1.** Characteristics of the study participants classified by serum uric acid levels.

Variable	All participants median (p25-p75) *n* (%)	With hyperuricemia median (p25-p75) *n*(%)	With normal uricemia median (p25-p75) *n*(%)	*P* value
Total Men Women	860 (100) 448 (52) 412 (48)	129 (15) 88 (19.6) 41 (10)	731 (85) 360 (80.4) 371 (90)	<0.001 ^a^
Age (years)	19.0 (18.0-20.0)	19.0 (18.0-20.0)	19.0 (18.0-20.0)	0.138 ^b^
BMI (Kg/m ^2^)	23.03 (20.56-26.58)	25.7 (23.0-29.7)	22.6 (20.2-26.1)	<0.001 ^b^
Systolic pressure (mmHg)	110 (100-110)	110 (100-120)	110 (100-110)	<0.001 ^b^
Diastolic pressure (mmHg)	70 (60-70)	70(70-80)	70 (60-70)	<0.001 ^b^
Glucose (mg/dL)	79.0 (74.5-84.0)	81.0 (75.0-86.0)	79.0 (74.0-84.0)	0.087 ^b^
Uric acid (mg/dL)	5.3 (4.2-6.2)	7.6 (6.9-8.2)	4.9 (4.1-5.7)	<0.001 ^b^
Triglycerides (mg/dL)	96.0 (71.0-133.0)	126.0 (96.0-178.0)	90.0 (68.0-126.5)	<0.001 ^b^
Total Cholesterol (mg/dL)	150.0 (131.0-170.0)	164.0 (144.0-188.5)	146.0 (128.0-166.0)	<0.001 ^b^
LDL Cholesterol (mg/dL)	58.5 (40.8-70.05)	69.1 (49.7-94.3)	57.1 (40.2-73.0)	<0.001 ^b^
HDL Cholesterol (mg/dL)	67.9 (58.8-76.9)	64.8 (56.1-74.9)	68.3 (59.3-76.9)	0.083 ^b^
*ABCG2* (Q191K) Alleles G T Genotypes GG GT TT	1298 (75) 422 (25) 490 (57) 318 (37) 52 (6)	183 (71) 75 (29) 64 (49.6) 55 (42.5) 10 (7.9)	1115 (76) 347 (24) 426 (58) 263 (36) 42 (6)	0.056 ^a^ 0.17 ^a^
*SLC22A12* (517G>A) Alleles G A Genotypes GG GA AA	558 (32) 1162 (68) 94 (11) 370 (43) 396 (46)	84 (33) 174 (67) 15 (11.8) 54 (41.7) 60 (46.5)	474 (32) 988 (68) 79 (11) 316 (43) 336 (46)	0.9 ^a^ 0.94 ^a^
*XDH* (518T>C) Alleles T C Genotypes TT TC CC	1083 (63) 637 (37) 335 (39) 413 (48) 112 (13)	167 (65) 91 (35) 53 (41) 61 (47.2) 15 (11.8)	916 (63) 546 (37) 282 (39) 352 (48) 97 (13)	0.52 ^a^ 0.8 ^a^

Abbreviations: BMI, body mass index; *ABCG2,* gene that encodes an ATP-binding cassette transporter subfamily G member 2; *SLC22A12,* gene that encodes an urate transporter 1 gene; *XDH,* gene that encodes a xanthine dehydrogenase. ^a^Chi-square test. ^b^Mann‒Whitney *U* test. ^c^Serum uric acid ˃6 and 7 mg/dL was considered as hyperuricemia for women and men, respectively. Data are shown as median and 25th and 75th percentile range (p25–p75). The level of significance was set at *p* <0.05.

**Table 2.** Allele and genotype frequencies between male and female participants.

	Men *n*(%)	Women *n* (%)	*P* value ^a^
*ABCG2* (Q191K) Alleles G T Genotypes GG GT TT	683 (76) 213 (24) 255 (57) 173 (39) 20 (4)	615 (75) 209 (25) 233 (57) 149 (36) 30 (7)	0.44 0.19
*SLC22A12* (517G>A) Alleles G A Genotypes GG GA AA	286 (32) 610 (68) 47 (10) 192 (43) 209 (47)	272 (33) 552 (67) 49 (12) 174 (42) 189 (46)	0.63 0.80
*XDH* (518T>C) Alleles T C Genotypes TT TC CC	567 (63) 329 (37) 181 (40) 205 (46) 62 (14)	516 (63) 308 (37) 156 (38) 204 (50) 52 (12)	0.77 0.54

Abbreviations: *ABCG2*, gene that encodes an ATP-binding cassette transporter subfamily G member 2; *SLC22A12,* gene that encodes an urate transporter 1 gene; *XDH*, gene that encodes a xanthine dehydrogenase. ^a^Chi-square test. The level of significance was set at *p* <0.05.

**Figure 1.**
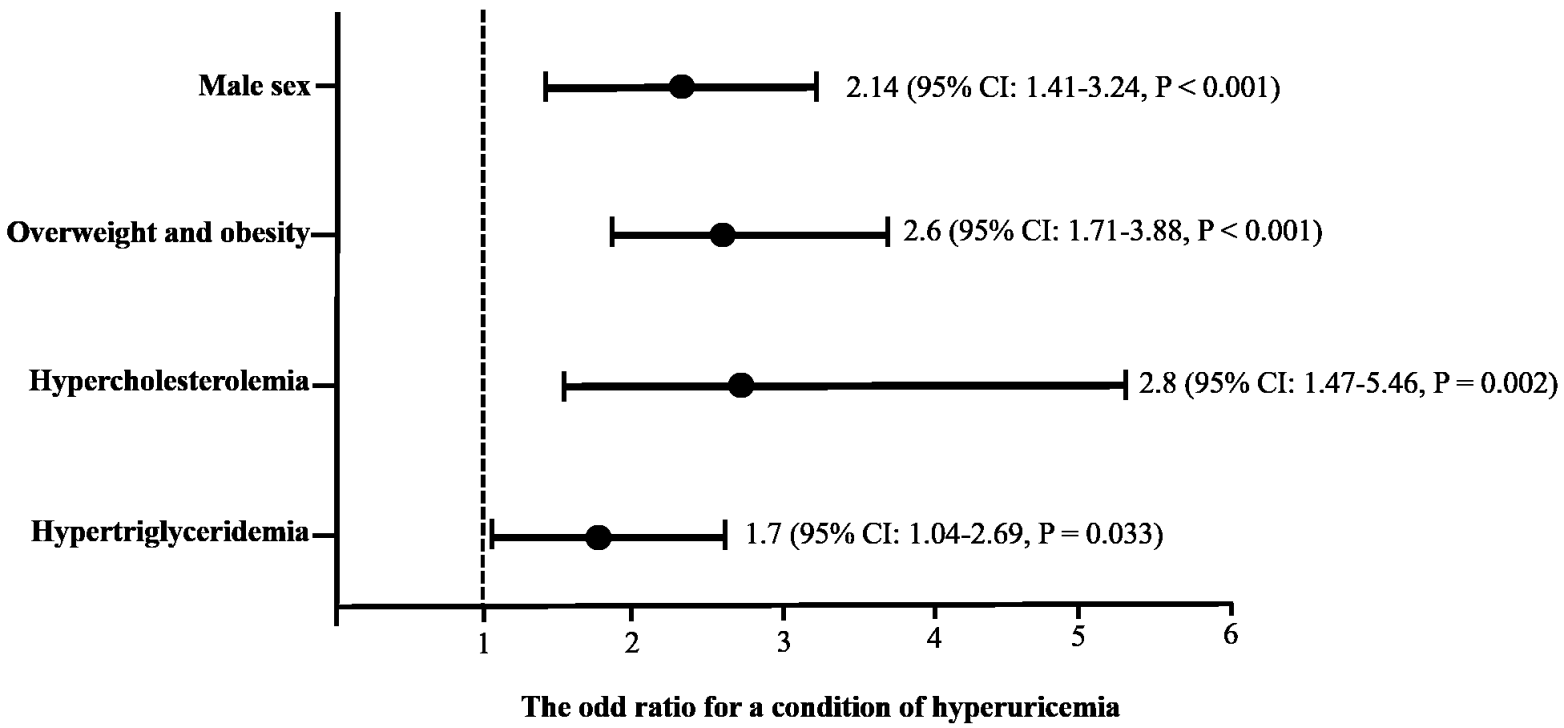
Association of sex, body mass index and dyslipidemias as predictive factors of hiperuricemia using a multivariate logistic regression analysis. The cutoff criteria were based on the World Health Organization and the National Cholesterol Education Program Adult (ATP III). Overweight: body mass index ≥ 25, Obesity: body mass index ˃30; hypercholesterolemia: fasting serum cholesterol levels ˃ 200 mg/dL; hypertriglyceridemia: fasting serum triglycerides levels ˃ 150 mg/dL.

Considering the statistically significant differences among the variables, a multivariate logistic regression analysis was performed, including sex, BMI, hypercholesterolemia, and hypertriglyceridemia as predictors of hyperuricemia ( Figure 1). A predisposition to abnormal serum uric acid levels was found in some conditions as follows: male sex (odds ratio (OR): 2.14, 95% confidence interval (CI): 1.41-3.24, *p* = ˂ 0.001); overweight or obese (OR: 2.6, 95% CI: 1.71-3.88, *p* = ˂0.001); having hypercholesterolemia (OR: 2.8, 95% CI: 1.47-5.46, *p* = 0.002); or having hypertriglyceridemia (OR: 1.7, 95% CI: 1.04-2.69, *p* = 0.033).

In Table 3, an analysis by genotype is shown for the *ABCG2* (Q191K), *SLC22A12* (r517G >A), and *XDH* (518T>C) polymorphisms, employing a dominant model for all three. With respect to the *ABCG2* (Q191K) polymorphism, statistically significant differences were found between the GT+TT genotypes vs. GG for serum uric acid ( *p* = 0.003) total cholesterol ( *p* = 0.005) and HDL-C levels ( *p* = 0.003). GA+AA genotypes vs. GG of the *SLC22A12* (517G>A) polymorphism only showed to influence LDL-C levels ( *p* = 0.043). Finally, no statistically significant differences were found by genotype for the *XDH* (518T>C) polymorphism ( *p* > 0.05).

**Table 3.** Analysis of variables by genotype for the *ABCG2* (Q191K), *SLC22A12* (517G>A), and *XDH* (518T>C) genetic polymorphisms.

Variable	*ABCG2* (Q191K) Dominant model		*P*-Value ^a^
	GT+TT median (25th-75th percentile range)	GG median (25th-75th percentile range)	
BMI (Kg/m ^2^)	23.1 (20.4 – 27.0)	22.7 (20.2 – 26.3)	0.393
Systolic pressure (mmHg)	110 (100.0 – 110.0)	110 (100.0 – 110.0)	0.632
Diastolic pressure (mmHg)	70.0 (60.0 – 70.0)	70.0 (60.0 – 70.0)	0.220
Glucose (mg/dL)	80.0 (76.0 – 85.0)	80.0 (75.0 – 84.0)	0.129
Uric Acid (mg/dL)	5.00 (4.2 – 6.2)	4.7 (3.9 – 5.4)	**0.003**
Triglycerides (mg/dL)	94.0 (69.5 – 134.0)	92.0 (68.0 – 127.0)	0.634
Total Cholesterol (mg/dL)	151 (132.0 – 173.0)	145 (125.0 – 164.0)	**0.005**
LDL Cholesterol (mg/dL)	59.2 (42.5 – 74.8)	53.8 (38.8 – 72.7)	0.070
HDL Cholesterol (mg/dL)	70.9 (62.3 – 78.5)	67.3 (57.8 – 76.8)	**0.003**
	*SLC22A12* (517G>A) Dominant model		
	GA+AA median (p25-p75)	GG median (p25-p75)	
BMI (Kg/m ^2^)	22.6 (20.1–26.2)	23.2 (20.4–26.6)	0.136
Systolic pressure (mmHg)	110.0 (100.0 – 110.0)	110.0 (100.0 – 110.0)	0.662
Diastolic pressure (mmHg)	70.0 (60.0 – 70.0)	70.0 (60.0 – 70.0)	0.600
Glucose (mg/dL)	80.0 (76.0 – 85.0)	80.0 (75.0 – 84.5)	0.302
Uric Acid (mg/dL)	4.8 (4.0 – 5.5)	4.90 (4.1 – 5.6)	0.537
Triglycerides (mg/dL)	91.0 (68.0 – 135.0)	94.5 (70.0 – 125.5)	0.763
Total Cholesterol (mg/dL)	144.0 (126.0 – 167.0)	149.5 (131.5 – 169.0)	0.127
LDL Cholesterol (mg/dL)	54.3 (36.1 – 70.5)	58.4 (40.6 – 75.2)	**0.043**
HDL Cholesterol (mg/dL)	69.3 (59.1 – 77.6)	68.1 (59.8 – 77.1)	0.659
	*XDH* (518T>C) Dominant model		
	TC+CC median (p25-p75)	TT median (p25-p75)	
BMI (Kg/m ^2^)	22.8 (20.2–26.4)	23.0 (20.4–26.6)	0.618
Systolic pressure (mmHg)	110.0 (100.0 – 110.0)	110.0 (100.0 – 110.0)	0.460
Diastolic pressure (mmHg)	70.0 (60.0 – 70.0)	70.0 (60.0 – 70.0)	0.949
Glucose (mg/dL)	80.0 (75.0 – 85.0)	79.5 (76.0 – 84.0)	0.521
Uric Acid (mg/dL)	4.80 (4.0 – 5.5)	4.90 (4.0 – 5.5)	0.840
Triglycerides (mg/dL)	94 (69.0 – 128.5)	91.0 (68.0 – 132.0)	0.944
Total Cholesterol (mg/dL)	147.0 (128.0 – 168.0)	145.5 (129.0 – 167.0)	0.564
LDL Cholesterol (mg/dL)	56.6 (39.5 – 73.3)	56.1 (39.1 – 72.9)	0.640
HDL Cholesterol (mg/dL)	69.1 (60.1 – 77.6)	67.4 (58.9 – 76.9)	0.370

Abbreviations: BMI, body mass index; *ABCG2,* gene that encodes an ATP-binding cassette transporter subfamily G member 2; *SLC22A12,* gene that encodes an urate transporter 1 gene; *XDH,* gene that encodes a xanthine dehydrogenase. ^a^Mann‒Whitney *U* test. The level of significance was set at *p* <0.05.

When the OR and the association between genotypes for the *BCG2* (Q191K) and *SLC22A12* (517G>A) polymorphisms and cardiovascular risks were calculated by sex, some significant results were found utilizing a dominant model. Table 4 reports that GT+TT genotypes for the *ABCG2* (Q191K) polymorphism significantly statistically increases the risk of hyperuricemia (OR = 2.43, 95% CI: 1.41-4.17, *p* = 0.001) and hypercholesterolemia (OR = 4.89, 95% CI: 1.54-15.48, *p* = 0.003), but only in male participants. On the other hand, no significant results were found for the genotypes of the *SLC22A12* (517G>A) and *XDH* (518T>C) polymorphisms.

**Table 4.** Analyses for the *ABCG2* (Q191K), *SLC22A12* (517G>A), and *XDH* (518T>C) polymorphisms and risk associated by sex.

Condition ^a^	Dominant model for the *ABCG2* (Q191K) polymorphism GT+TT vs. GG					
	All participants		Men		Women	
	OR (95% CI)	*P* value	OR (95% CI)	*P* value	OR (95% CI)	*P* value
Overweight and obesity	1.10 (0.78-1.56)	0.559	1.57 (0.93-2.67)	0.089	0.84 (0.53-1.34)	0.481
SP ˃130 mmHg	1.15 (0.38-3.47)	0.799	2.81 (0.68-11.52)	0.135	0.32 (0.03-2.97)	0.298
DP ˃85 mmHg	0.33 (0.03-2.99)	0.303	1.35 (0.08-21.94)	0.829	0.44 (0.04-4.28)	0.468
Fasting glucose ˃100 mg/dL	4.1 (0.82-20.49)	0.063	2.74 (0.24-30.63)	0.394	5.4 (0.60-49.39)	0.091
Hyperuricemia	**1.49 (1.00-2.21)**	**0.047**	**2.43 (1.41-4.17)**	**0.001**	0.77 (0.39-1.53)	0.465
Hypertriglyceridemia	0.84 (0.54-1.31)	0.463	1.32 (0.76-2.30)	0.315	0.87 (0.50-1.51)	0.635
Hypercholesterolemia	**3.27 (1.62-6.59)**	**0.001**	**4.89 (1.54-15.48)**	**0.003**	2.09 (0.83-5.26)	0.110
LDL-C ≥130 mg/dL	3.1 (0.81-12.47)	0.078	5.59 (0.61-50.80)	0.086	2.0 (0.33-12.26)	0.435
Low HDL-C	0.37 (0.07-1.83)	0.210	0.72 (0.319-1.644)	0.440	0.44 (0.045-4.2)	0.468
Condition ^a^	Dominant model for the *SLC22A12* (517G>A) polymorphism GA+AA vs. GG					
	All participants		Men		Women	
	OR (95% CI)	*P* value	OR (95% CI)	*P* value	OR (95% CI)	*P* value
Overweight and obesity	0.81 (0.57-1.14)	0.232	0.72 (0.42-1.22)	0.227	0.88 (0.56-1.39)	0.599
SP ˃130 mmHg	1.01 (0.33-3.04)	0.986	1.09 (0.28-4.19)	0.892	0.85 (0.11-6.15)	0.878
DP ˃85 mmHg	1.30 (0.21-7.84)	0.773	1.76 (0.15-19.69)	0.641	0.42 (0.03-4.74)	0.475
Fasting glucose ˃100 mg/dL	2.6 (0.52-13.13)	0.222	0.43 (0.03-4.84)	0.485	4.3 (0.50-37.99)	0.143
Hyperuricemia	0.99 (0.67-1.48)	0.988	1.23 (0.66-1.90)	0.667	0.84 (0.43-1.62)	0.607
Hypertriglyceridemia	1.15 (0.74-1.78)	0.515	1.32 (0.76-2.29)	0.322	1.25 (0.72-2.15)	0.417
Hypercholesterolemia	1.26 (0.65-2.44)	0.486	1.66 (0.59-4.64)	0.330	0.85 (0.34-2.10)	0.725
LDL-C ≥130 mg/dL	1.30 (0.36-4.67)	0.682	3.58 (0.39-35.51)	0.227	0.56 (0.09-3.44)	0.533
Low HDL-C	0.68 (0.18-2.56)	0.579	1.011 (0.045-2.22)	0.979	0.42 (0.03-4.74)	0.475
Condition ^a^	Dominant model for the *XDH* (518T>C) polymorphism TC+CC vs. TT					
	All participants		Men		Women	
	OR (95% CI)	*P* value	OR (95% CI)	*P* value	OR (95% CI)	*P* value
Overweight and obesity	0.97 (0.68-1.38)	0.886	1.17 (0.68-1.99)	0.559	0.85 (0.53-1.36)	0.503
SP ˃130 mmHg	0.74 (0.24-2.23)	0.594	0.85 (0.22-3.27)	0.882	0.60 (0.08-4.23)	0.609
DP ˃85 mmHg	0.95 (0.15-5.78)	0.964	0.68 (0.04-11.13)	0.791	1.2 (0.10-13.50)	0.876
Fasting glucose ˃100 mg/dL	4.5 (0.55-37.23)	0.122	1.38 (0.12-15.49)	0.790	3.0 (0.35-26.63)	0.284
Hyperuricemia	0.90 (0.63-1.34)	0.613	0.77 (0.45-1.31)	0.342	1.29 (0.64-2.61)	0.470
Hypertriglyceridemia	0.84 (0.54-1.30)	0.444	0.68 (0.39-1.18)	0.174	1.41 (0.80-2.51)	0.230
Hypercholesterolemia	1.30 (0.65-2.59)	0.451	1.28 (0.46-3.60)	0.630	1.44 (0.53-3.85)	0.464
LDL-C ≥130 mg/dL	1.50 (0.38-5.87)	0.555	1.03 (0.17-6.31)	0.969	2.4 (0.27-22.15)	0.411
Low HDL-C	1.28 (0.31-5.18)	0.725	0.722 (0.350-1.70)	0.522	1.8 (0.18-17.75)	0.598

Abbreviations: BMI, body mass index; SP, systolic pressure; DP, diastolic pressure. LDL-C, LDL cholesterol; HDL-C, HDL cholesterol; *ABCG2,* gene that encodes an ATP-binding cassette transporter subfamily G member 2; *SLC22A12,* gene that encodes an urate transporter 1 gene; *XDH,* gene that encodes a xanthine dehydrogenase. ^a^The cut-off criteria were according to The World Health Organization and the National Cholesterol Education Program Adult (ATP III). Overweight was defined as BMI ≥25, while Obesity was BMI ˃30; Hyperuricemia was defined as serum uric acid ˃6 and 7 mg/dL for women and men, respectively; hypertriglyceridemia as fasting serum triglycerides ˃150 mg/dL, hypercholesterolemia as fasting serum cholesterol ˃200 mg/dL, and low HDL-C as a serum concentration of ˂50 mg/dL and 40 mg/dL for women and men, respectively. Multivariate logistic regression analysis: data are shown as odd ratio (95% Confidence Interval). The level of significance was set at *p* <0.05.

## Discussion

A total of 860 college applicants between the ages of 18 and 25 years of both sexes were included in this study, an important age range to identify early disorders. Indeed, we found a 15% prevalence of hyperuricemia, being higher among males (19.6%) than females (10%), as previously observed in another study with comparable age groups in Mexico ( Alegría-Díaz *et al.*, 2018). Regarding uric acid levels, similar blood concentrations have also been reported in Mexican young persons ( Pérez-Navarro *et al.*, 2020). The subgroup with hyperuricemia was prone to higher levels of BMI, blood pressure, triglycerides, total cholesterol, and LDL cholesterol ( Table 1). In fact, the association between serum uric acid levels and the traits of metabolic syndrome has been previously analyzed ( Lin *et al.*, 2006; Zhang *et al.*, 2020a), although some authors found no causal evidence of uric acid levels being associated with metabolic syndrome and its components ( Wang *et al.*, 2020). According to our results, being a man, being overweight or obese, or having dyslipidemia are related to high uric acid levels ( Figure 1). Men have a lower capacity to eliminate urate via the kidney compared with females due to a deficiency of estrogen and progesterone ( Hak *et al*., 2010). Likewise, the involvement of uric acid in lipid metabolism can lead to hyperuricemia, a condition considered as predictor factor of dyslipidemia ( Kuwabara *et al*. 2020; Lima *et al.*, 2015; Son *et al.*, 2016) and can subsequently alter blood pressure ( Teng *et al.*, 2011; Zhang *et al*., 2020a). In this way, our results summarized in Figure 1 show that being male, being obese and having dyslipidemia increases the risk of hyperuricemia ( Liu *et al.*, 2020a).

The study of the genetic influence on blood uric acid levels has included the search for single nucleotide polymorphisms (SNP) in genes involved in purine metabolism and urate removal. Here, we analyzed three polymorphisms in three different genes, including *ABCG2* (Q191K), *SLC22A12* (517G>A), and *XDH* (518T>C). The first corresponds to the exchange of a glutamine for a lysine at position 141 of an adenosine triphosphate (ATP)-binding cassette transporter subfamily G member 2 ( *ABCG2*). This exchange predisposes individuals to hyperuricemia ( Nakashima *et al.*, 2020; Wrigley *et al*., 2020). A frequency of 0.25 for the risk for T allele was found, a slightly lower prevalence than that reported in Asian and New Zealand populations ( Kim *et al.*, 2015; Liu *et al.*, 2020b; Toyoda *et al.*, 2019). The distribution of the T allele was marginal between hyperuricemic and normouricemic groups, being more frequent in the hyperuricemic group ( *p* = 0.056). When a dominant model was carried out, the risk for the T allele was associated with hyperuricemia and hypercholesterolemia (total and HDL cholesterol). This association was confirmed only in men, showing that the *ABCG2* (Q191K) polymorphism increases the risk for hyperuricemia 2.43 times (95% CI: 1.41-4.17, *p* = 0.001) and hypercholesterolemia 4.89 times (95% CI: 1.54-15.48, *p* = 0.003) in a dominant model. Other studies have also found a greater influence of this polymorphism in men ( Narang *et al.*, 2019) although, under some conditions, the *ABCG2* (Q191K) polymorphism could contribute to increased uric acid in women ( Guo *et al.*, 2020; Roman *et al.*, 2020). Although initially our work focused on hyperuricemia in young people, the increase in serum cholesterol in men associated with the *ABCG2* (Q191K) polymorphism was observed. There are still few studies linking the *ABCG2* gene and cholesterolemia. A relevant fact is that mRNA expression levels of *ABCG2* appear to be higher in individuals with hypercholesterolemia ( Rodrigues *et al.*, 2009); likewise, the activity of ABCG2 has been associated with cholesterol levels both *in vitro* and *in vivo (* To *et al.*, 2014). Therefore, perform genotype-based mRNA expression analysis to further explore the role of the *ABCG2* (Q191K) polymorphism in hyperuricemia and hypercholesterolemia should be contemplated.

The *SLC22A12* (517G>A) polymorphism comprises the transition from guanine to adenine in the 3´-UTR region in the urate transporter 1 gene; this transition appears to modify uric acid levels ( Flynn *et al.*, 2013; Köttgen *et al.*, 2013). In this study, the frequency of the minor allele was 0.32; however, there was no statistically different distribution of this between hyperuricemic and normouricemic groups. Although no differences in uric acid levels were associated with this polymorphism, lower LDL cholesterol levels were observed in the group of carriers of the A allele when data were analyzed in a dominant model ( *p* = 0.043), suggesting a protective effect of this allele ( Simon *et al.*, 2014). Since the *SLC22A12* (517G>A) polymorphism is located at a potential miRNA binding site ( Flynn *et al*., 2013), the epigenetic mechanism involved in the regulation of uric acid levels needs to be studied.

With respect to the *XDH* (518T>C) polymorphism, we studied the xanthine dehydrogenase polymorphism located in the 3´-UnTRanslated (UTR) region of the *XDH* gene. The replacement of T by C is considered a risk factor related to hypertension ( Wu *et al.*, 2015). We found that the minor allele frequency was 0.37 for the C allele, contrary to what was reported in a Chinese population, where the C allele had a higher frequency, in addition to its being associated with hypertension ( Wu *et al.*, 2015). However, the link between the *XDH* (518T>C) polymorphism and hypertension was not demostrated in Taiwanese women ( Lee *et al.*, 2019). In the present study, we did not find a statistically significant different distribution of the C allele between the hyperuricemic and normouricemic groups, and the risk allele was also not associated with any component of the metabolic syndrome.

Finally, hyperuricemia is an undesirable condition that has been seen as a minor trait of metabolic syndrome, cardiovascular risk, as well as other types of disorders such as psoriasis and alopecia, which have been associated with high levels of blood uric acid ( Guo *et al.*, 2020; Ma *et al.*, 2020; Talebi *et al*., 2020; Zhang *et al.*, 2020a). Even though our criterion for hyperuricemia were defined as blood uric acid >6 mg/dL for women and 7 mg/dL for men, cut-off points could be reconsidered according to the considerations made by Alegría-Díaz *et al.* (2018). In this regard, a recent study considers uric acid levels below 5 mg/dL for men and below 2-4 mg/dL for women to be optimal for a lesser risk of cardiometabolic diseases in a Japanese population ( Kuwabara *et al.*, 2020). Although there are modifiable factors related to lifestyle, genetic inheritance plays a decisive role in the control of uricemia.

This study has three main limitations: i) only one polymorphism was genotyped for every gene, analysis of multiple SNPs by haplotypes could be more appropriate; ii) the analysis of the *ABCG2* (Q191K) polymorphism is limited by the small sample size and the compromised statistical power; and, iii) new cut-off values for hyperuricemia have been suggested ( Alegría-Díaz *et al.*, 2018); however the conservative criteria of >6 mg/dL for women and 7 mg/dL for men were considered in this study.

## Conclusions

In this study, we found that the *ABCG2* (Q191K) polymorphism increases the risk of hyperuricemia as well as of hypercholesterolemia in young Mexican males. Since the *ABCG2* (Q191K) polymorphism can modify the efficacy of statins in reducing cholesterol ( Zhang *et al.*, 2020b), carriers of the risk allele represent a vulnerable group of interest for future pharmacogenetic research. Some considerations for future studies are including lifestyle and diet factors, the monitoring of the study population, and exploring more polymorphisms in the *ABCG2*, *SLC22A12*, and *XDH* genes, as well as studying haplotypes.

## Data availability

### Underlying data

Data mendeley: Polymorphisms of the genes ABCG2, SLC22A12 and XDH and their relation with hyperuricemia and hypercholesterolemia in Mexican young adults. http://dx.doi.org/10.17632/243ft29b7m.1 ( Alegría-Torres, 2021).

This project contains the following underlying data:

- DATABASE ART ARCHIVES OF PHYSIOL AND BIOCHEM.sav (spreadsheet of participant data)

Data are available under the terms of the Creative Commons Attribution 4.0 International license (CC-BY 4.0).

## References

[ref-1] Alegría-Díaz A Valdez-Ortiz R Murguía-Romero M: Clinical significance of serum uric acid levels in Mexican young adults. *Contrib Nephrol.* 2018;192:125–134. 10.1159/000484287 29393152

[ref-2] Alegría-Torres J: Polymorphisms of the genes ABCG2, SLC22A12 and XDH and their relation with hyperuricemia and hypercholesterolemia in Mexican young adults.Mendeley Data, V1,2021. 10.17632/243ft29b7m.1 PMC8474103 34631016

[ref-3] Bardin T Richette P: Definition of hyperuricemia and gouty conditions. *Curr Opin Rheumatol.* 2014;26(2):186–191. 10.1097/BOR.0000000000000028 24419750

[ref-4] Caulfield MJ Munroe PB O'Neill D: SLC2A9 is a high-capacity urate transporter in humans. *PLoS Med.* 2008;5(10):e197. 10.1371/journal.pmed.0050197 18842065 PMC2561076

[ref-5] Flynn TJ Phipps-Green A Hollis-Moffatt JE: Association analysis of the *SLC22A11* (organic anion transporter 4) and *SLC22A12* (urate transporter 1) urate transporter locus with gout in New Zealand case-control sample sets reveals multiple ancestral-specific effects. *Arthritis Res Ther.* 2013;15(6):R220. 10.1186/ar4417 24360580 PMC3978909

[ref-6] Galán I Goicoechea M Quiroga B: Hyperuricemia is associated with progression of chronic kidney disease in patients with reduced functioning kidney mass. *Nefrologia (Engl Ed).* 2018;38(1):73–78. 10.1016/j.nefro.2017.04.006 28869042

[ref-7] Guo G Huang Z Wang S: Sex differences in uric acid and NT-pro BNP assessments during coronary severity. *Medicine (Baltimore).* 2020;99(15):e19653. 10.1097/MD.0000000000019653 32282714 PMC7220359

[ref-8] Hak AE Curhan GC Grodstein F: Menopause, postmenopausal hormone use and risk of incident gout. *Ann Rheum Dis.* 2010;69(7):1305–1319. 10.1136/ard.2009.109884 19592386 PMC3142742

[ref-9] Harrison R: Structure and function of xanthine oxidoreductase: where are we now? *Free Radic Biol Med.* 2002;33(6):774–797. 10.1016/s0891-5849(02)00956-5 12208366

[ref-10] Hsu CY Iribarren C McCulloch CE: Risk factors for end-stage renal disease: 25-year follow-up. *Arch Intern Med.* 2009;169(4):342–350. 10.1001/archinternmed.2008.605 19237717 PMC2727643

[ref-11] Kim YS Kim Y Park G: Genetic analysis of *ABCG2* and *SLC2A9* gene polymorphisms in gouty arthritis in a Korean population. *Korean J Intern Med.* 2015;30(6):913–920. 10.3904/kjim.2015.30.6.913 26552468 PMC4642022

[ref-12] Köttgen A Albrecht E Teumer A: Genome-wide association analyses identify 18 new loci associated with serum urate concentrations. *Nat Genet.* 2013;45(2):145–154. 10.1038/ng.2500 23263486 PMC3663712

[ref-13] Kuwabara M Hisatome I Niwa K: The optimal range of serum uric acid for cardiometabolic diseases: a 5-year Japanese cohort study. *J Clin Med.* 2020;9(4):942. 10.3390/jcm9040942 32235468 PMC7231289

[ref-14] Lee JH Go TH Lee SH: Association between serum urate and risk of hypertension in menopausal women with XDH gene. *J Clin Med.* 2019;8(5):738. 10.3390/jcm8050738 31126092 PMC6571698

[ref-15] Lima WG Martins-Santos MES Chaves VE: Uric acid as a modulator of glucose and lipid metabolism. *Biochimie.* 2015;116:17–23. 10.1016/j.biochi.2015.06.025 26133655

[ref-16] Lin SD Tsai DH Hsu SR: Association between serum uric acid level and components of the metabolic syndrome. *J Chin Med Assoc.* 2006;69(11):512–516. 10.1016/S1726-4901(09)70320-X 17116612

[ref-17] Liu F Du GL Song N: Hyperuricemia and its association with adiposity and dyslipidemia in Northwest China: results from cardiovascular risk survey in Xinjiang (CRS 2008-2012). *Lipids Health Dis.* 2020a;19(1):58. 10.1186/s12944-020-01211-z 32238146 PMC7115071

[ref-18] Liu J Yang W Li Y: *ABCG2* rs2231142 variant in hyperuricemia is modified by *SLC2A9* and *SLC22A12* polymorphisms and cardiovascular risk factors in an elderly community-dwelling population. *BMC Med Genet.* 2020b;21(1):54. 10.1186/s12881-020-0987-4 32183743 PMC7077001

[ref-19] Ma J Sheng Y Lao Z: Hyperuricemia is associated with androgenetic alopecia in men: a cross-sectional case-control study. *J Cosmet Dermatol.* 2020;19(11):3122–3126. 10.1111/jocd.13401 32281237

[ref-20] Macías-Kauffer LR Villamil-Ramírez H León-Mimila P: Genetic contributors to serum uric acid levels in Mexicans and their effect on premature coronary artery disease. *Int J Cardiol.* 2019;279:168–173. 10.1016/j.ijcard.2018.09.107 30305239

[ref-21] Martínez-Quintana E Tugores A Rodríguez-González F: Serum uric acid levels and cardiovascular disease: the Gordian knot. *J Thorac Dis.* 2016;8(11):E1462–E1466. 10.21037/jtd.2016.11.39 28066631 PMC5179380

[ref-22] Nakashima A Ichida K Ohkido I: Dysfunctional *ABCG2* gene polymorphisms are associated with serum uric acid levels and all-cause mortality in hemodialysis patients. *Hum Cell.* 2020;33(3):559–568. 10.1007/s13577-020-00342-w 32180207 PMC7324430

[ref-23] Narang RK Topless R Cadzow M: Interactions between serum urate-associated genetic variants and sex on gout risk: analysis of the UK Biobank. *Arthritis Res Ther.* 2019;21(1):13. 10.1186/s13075-018-1787-5 30626429 PMC6327586

[ref-24] Pérez-Navarro LM Valdez-Ortiz R Alegría-Díaz A: CARDIOMETABOLIC RISK FACTORS ASSOCIATED WITH RENAL FUNCTION IN APPARENTLY HEALTHY YOUNG STUDENTS: A CROSS-SECTIONAL STUDY. *Rev Invest Clin.* 2020;72(2):95–102. 10.24875/RIC.19003204 32284621

[ref-25] Rivera-Paredez B Macías-Kauffer L Fernandez-Lopez JC: Influence of genetic and non-genetic risk factors for serum uric acid levels and hyperuricemia in Mexicans. *Nutrients.* 2019;11(6):1336. 10.3390/nu11061336 31207883 PMC6627998

[ref-26] Rodrigues AC Hirata MH Hirata RDC: Impact of cholesterol on ABC and SLC transporters expression and function and its role in disposition variability to lipid-lowering drugs. *Pharmacogenomics.* 2009;10(6):1007–1016. 10.2217/pgs.09.18 19530968

[ref-27] Roman Y Tiirikainen M Prom-Wormley E: The prevalence of the gout-associated polymorphism rs2231142 G>T in *ABCG2* in a pregnant female Filipino cohort. *Clin Rheumatol.* 2020;39(8):2387–2392. 10.1007/s10067-020-04994-9 32107664

[ref-28] Simon KC Eberly S Gao X: Mendelian randomization of serum urate and Parkinson disease progression. *Ann Neurol.* 2014;76(6):862–868. 10.1002/ana.24281 25257975 PMC4245314

[ref-29] Son M Seo J Yang S: Association between dyslipidemia and serum uric acid levels in Korean adults: Korea National Health and Nutrition Examination Survey 2016-2017. *PLoS One.* 2016;15(2):e0228684. 10.1371/journal.pone.0228684 32059030 PMC7021293

[ref-30] Talebi A Amirabadizadeh A Nakhaee S: Cerebrovascular disease: how serum phosphorus, vitamin D, and uric acid levels contribute to the ischemic stroke. *BMC Neurol.* 2020;20(1):116. 10.1186/s12883-020-01686-4 32234035 PMC7110613

[ref-31] Teng F Zhu R Zou C: Interaction between serum uric acid and triglycerides in relation to blood pressure. *J Hum Hypertens.* 2011;25(11):686–691. 10.1038/jhh.2010.112 21160529

[ref-32] To KK Hu M Tomlinson B: Expression and activity of ABCG2, but not ABCB1 or OATP1B1, are associated with cholesterol levels: evidence from *in vitro* and *in vivo* experiments. *Pharmacogenomics.* 2014;15(8):1091–1014. 10.2217/pgs.14.58 25084202

[ref-33] Toyoda Y Mančíková A Krylov V: Functional characterization of clinically-relevant rare variants in *ABCG2* identified in a gout and hyperuricemia cohort. *Cells.* 2019;8(4):363. 10.3390/cells8040363 31003562 PMC6523779

[ref-34] Wang L Zhang T Liu Y: Association of serum uric acid with metabolic syndrome and its components: a Mendelian randomization analysis. *Biomed Res Int.* 2020;2020:6238693. 10.1155/2020/6238693 32258131 PMC7063870

[ref-35] Woodward OM Köttgen A Coresh J: Identification of a urate transporter, ABCG2, with a common functional polymorphism causing gout. *Proc Natl Acad Sci U S A.* 2009;106(25):10338–10342. 10.1073/pnas.0901249106 19506252 PMC2700910

[ref-36] Wrigley R Phipps-Green AJ Topless RK: Pleiotropic effect of the *ABCG2* gene in gout: involvement in serum urate levels and progression from hyperuricemia to gout. *Arthritis Res Ther.* 2020;22(1):45. 10.1186/s13075-020-2136-z 32164793 PMC7069001

[ref-37] Wu B Hao Y Shi J: Association between xanthine dehydrogenase tag single nucleotide polymorphisms and essential hypertension. *Mol Med Rep.* 2015;12(4):5685–5690. 10.3892/mmr.2015.4135 26239312 PMC4581766

[ref-38] Zhang D Ding Y Wang X: Effects of *ABCG2* and *SLCO1B1* gene variants on inflammation markers in patients with hypercholesterolemia and diabetes mellitus treated with rosuvastatin. *Eur J Clin Pharmacol.* 2020b;76(7):939–946. 10.1007/s00228-020-02882-4 32361904

[ref-39] Zhang L Li JL Guo LL: The interaction between serum uric acid and triglycerides level on blood pressure in middle-aged and elderly individuals in China: result from a large national cohort study. *BMC Cardiovasc Disord.* 2020a;20(1):174. 10.1186/s12872-020-01468-3 32293295 PMC7160924

[ref-40] Zuo T Liu X Jiang L: Hyperuricemia and coronary heart disease mortality: a meta-analysis of prospective cohort studies. *BMC Cardiovasc Disord.* 2016;16(1):207. 10.1186/s12872-016-0379-z 27793095 PMC5084405

