# Supplementary material for: Polymorphisms of the genes ABCG2, SLC22A12 and XDH and their relation with hyperuricemia and hypercholesterolemia in Mexican young adults
Source: F1000Res. 2021 Sep 24;10:217. Originally published 2021 Mar 17. [Version 2] doi: 10.12688/f1000research.46399.2 (PMC8474103; doi:10.12688/f1000research.46399.2)
Supplement: Peer review history

# Peer review: Reviewer response for version 2

**DOI:** 10.5256/f1000research.77844.r95320

**Published:** 2021-09-24

**Authors:** Jing He

**Affiliations:** 1 Department of Pediatric Surgery, Guangzhou Institute of Pediatrics, Guangdong Provincial Key Laboratory of Research in Structural Birth Defect Disease, Guangzhou Women and Children's Medical Center, Guangzhou Medical University, Guangzhou, China

Acceptable.

Is the work clearly and accurately presented and does it cite the current literature?

Partly

If applicable, is the statistical analysis and its interpretation appropriate?

Partly

Are all the source data underlying the results available to ensure full reproducibility?

Yes

Is the study design appropriate and is the work technically sound?

Yes

Are the conclusions drawn adequately supported by the results?

Yes

Are sufficient details of methods and analysis provided to allow replication by others?

Yes

Reviewer Expertise:

Molecular Epidemiology.

I confirm that I have read this submission and believe that I have an appropriate level of expertise to confirm that it is of an acceptable scientific standard.

# Peer review: Reviewer response for version 1

**DOI:** 10.5256/f1000research.49473.r91797

**Published:** 2021-08-24

**Authors:** Jing He

**Affiliations:** 1 Department of Pediatric Surgery, Guangzhou Institute of Pediatrics, Guangdong Provincial Key Laboratory of Research in Structural Birth Defect Disease, Guangzhou Women and Children's Medical Center, Guangzhou Medical University, Guangzhou, China

In the current study, the authors investigate the association between three polymorphisms in the *ABCG2*, *SLC22A12*, and *XDH *genes and hyperuricemia risk by including 860 subjects. They found the *ABCG2* rs2231142 polymorphism increases the risk of hyperuricemia and hypercholesterolemia in young Mexican males.

 I have some comments that should be addressed by the authors:

1. In the Abstract, it's better provide the number of cases and controls, as well as the nucleotide alterations for the polymorphisms they chose.
2. The authors only chose one polymorphism for each gene, that was not rationalised. It's better chose at least two potentially functional SNPs for each gene.
3. In the Materials and Methods, there was no need to provide the sequences of the probes. The authors should provide the function of the selected polymorphisms.
4. False-positive report probability and statistical power should be calculated as the positive findings for the samples included were so small (see the following articles, in which I have been involved with: He *et al.* Mol Carcinog. 2013; 52 Suppl 1: E70-9 ^1^ and He *et al. *Mol Ther Nucleic Acids. 2018; 11: 1-8 ^2^).
5. The authors found the *ABCG2* (rs2231142) polymorphism increases the risk of hyperuricemia and hypercholesterolemia in young Mexican males. It is lacking functional validation for the function of *ABCG2* (rs2231142) polymorphism. It’s better to perform genotype-based mRNA expression analysis to further explore the potential role of the significant polymorphism (He *et al.* Mol Ther Oncolytics, 2021; 20: 199-208 ^3^; Zhuo *et al. *Mol Ther Nucleic Acids, 2020; 22:17-26 ^4^). From these two references, which I have been involved with, the authors can perform genotype based mRNA expression analysis to further explore the potential role of the *ABCG2* (rs2231142) polymorphism.

Is the work clearly and accurately presented and does it cite the current literature?

Partly

If applicable, is the statistical analysis and its interpretation appropriate?

Partly

Are all the source data underlying the results available to ensure full reproducibility?

Yes

Is the study design appropriate and is the work technically sound?

Yes

Are the conclusions drawn adequately supported by the results?

Yes

Are sufficient details of methods and analysis provided to allow replication by others?

Yes

Reviewer Expertise:

Molecular Epidemiology.

I confirm that I have read this submission and believe that I have an appropriate level of expertise to confirm that it is of an acceptable scientific standard, however I have significant reservations, as outlined above.

## References

[1] : Genetic variations of mTORC1 genes and risk of gastric cancer in an Eastern Chinese population.*Mol Carcinog*.2013;52 Suppl 1: 10.1002/mc.22013E70-910.1002/mc.2201323423739

[2] : Association of Common Genetic Variants in Pre-microRNAs and Neuroblastoma Susceptibility: A Two-Center Study in Chinese Children.*Mol Ther Nucleic Acids*.2018;11: 10.1016/j.omtn.2018.01.0031-810.1016/j.omtn.2018.01.00329858046PMC5849804

[3] : Genetic variants in m6A modification core genes are associated with glioma risk in Chinese children.*Mol Ther Oncolytics*.2021;20: 10.1016/j.omto.2020.12.013199-20810.1016/j.omto.2020.12.01333665358PMC7889446

[4] : METTL14 Gene Polymorphisms Confer Neuroblastoma Susceptibility: An Eight-Center Case-Control Study.*Mol Ther Nucleic Acids*.2020;22: 10.1016/j.omtn.2020.08.00917-2610.1016/j.omtn.2020.08.00932891980PMC7484523

# Response

**Published:** 2021-09-21

**Authors:** Jorge Alegría

**Affiliations:** Universidad de Guanajuato, Mexico

Regarding the five points discussed, we respond to each of them:

 1. The number of the cases and controls as well as the description of sequence variants were included in the abstract.

 2. According to the introduction section, we selected three genes involved in the metabolism of purines or urates, which have versions that appear to predispose to a hyperuricemic condition; the ABCG2 gene that encodes a membrane transporter, which exports urates to the kidney and intestine (Woodward *et al*. 2009); and the SLC2A9 gene that encodes a kidney protein called GLUcose Transporter 9 (GLUT9), an important flow regulator of urates in the proximal tubules (Caulfield *et al.* 2008). In the same way, the XDH gene gives rise to an enzyme denominated xanthine dehydrogenase, which breaks down purines from nucleic acids, specifically the hypoxanthine‒xanthine‒urate conversion pathway (Harrison 2002). We chose polymorphisms in different genes for the following two reasons: 

1. Previous reports have shown the combined effect of genetic variants (specifically SLC2A9 and ABCG2) are able to explain 3-4% of hyperuricemia, modulated by gender and body mass index (Brandstätter *et al.*, 2010; Köttgen *et al.,* 2013; Huffman *et al.*, 2015 and Merriman *et al.*, 2015;). Further, the ABCG2 (Q191K) and SLC22A12 (517G>A) polymorophisms have been studied in Mexican population showing a high prevalence (Macías-Kauffer et al., 2019 and Rivera-Paredez *et al.* 2019). Besides, since the XDH (518T>C) polymorphism it has been poorly studied in Mexicans, we wanted to explore if it plays a role in hyperuricemia. 
2. We only analyzed polymorphisms with a minor allele frequency >5%, being: ABCG2 (Q191K) 25%, SLC22A12 (517G>A) 32%, and XDH (518T>C) 37%; as well as that the polymorphisms were in Hardy-Weinberg equilibrium. 

**References:**

 Brandstätter A, Lamina C, Kiechl S, Hunt SC, Coassin S, Paulweber B, *et al*. Sex and age interaction with genetic association of atherogenic uric acid concentrations. Atherosclerosis 2010; 210:474-8. 

 Huffman JE, Albrecht E, Teumer A, Mangino M, Kapur K, Johnson T, *et al.* Modulation of Genetic Associations with Serum Urate Levels by Body-Mass-Index in Humans. Crawford DC, editor. PLoS One 2015; 10:e0119752 

 Köttgen A, Albrecht E, Teumer A, Vitart V, Krumsiek J, Hundertmark C, *et al.* Genome-wide association analyses identify 18 new loci associated with serum urate concentrations. Nat Genet 2013; 45:145-54. 

 Merriman TR. An update on the genetic architecture of hyperuricemia and gout. Arthritis Res Ther 2015; 17:98.

 3. The sequences of the probes was deleted from the Materials and Methods section. The function of the selected polymorphisms is described in the discussion section: The ABCG2 (Q191K) polymorphism corresponds to the exchange of a glutamine for a lysine at position 141 of an adenosine triphosphate (ATP)-binding cassette transporter subfamily G member 2 (ABCG2). This exchange predisposes individuals to hyperuricemia (Nakashima *et al.* 2020, Wrigley *et al. *2020). The SLC22A12 (517G>A) polymorphism comprises the transition from guanin to adenine in the 3´-UTR region in the urate transporter 1 gene; this transition appears to modify uric acid levels (Flynn et al. 2013, Köttgen *et al.* 2013). With respect to the XDH (518T>C) polymorphism, the replacement of T by C is considered a risk factor related to hypertension (Wu *et al.* 2015).

 4. In fact, our results are impacted by the small sample size and the compromised statistical power. This point is discussed and included as a limitation of this study.

 5. This point is argued in the discussion section: There are still few studies linking the ABCG2 gene and cholesterolemia.

 A relevant fact is that mRNA expression levels of ABCG2 appear to be higher in individuals with hypercholesterolemia (Rodrigues *et al.* 2009); likewise, the activity of ABCG2 has been associated with cholesterol levels both in vitro and in vivo (To *et al.* 2014). Therefore, perform genotype-based mRNA expression analysis to further explore the role of the ABCG2 (Q191K) polymorphism in hyperuricemia and hypercholesterolemia should be contemplated.

# Peer review: Reviewer response for version 1

**DOI:** 10.5256/f1000research.49473.r81558

**Published:** 2021-03-25

**Authors:** Ilham Akbar Rahman

**Affiliations:** 1 Lecturer staff in Bachelor of Medicine Programme, Hasanuddin University, Makassar, Indonesia

The study assessed the relationship of ABCG2, SLC22A12, and XDH genes polymorphism on their relationship with hyperuricemia and risk factors of cardiovascular disease. It seems that the authors have provided a significantly huge amount of effort and cost in conducting this study. This polymorphism study could provide potential implications in the future on the prevention, diagnosis, and treatment of hyperuricemia associated with cardiovascular risk factors. The authors display the knowledge on good writing skills including grammar and word choice, sentence structure, and paragraph development. However, several points need to be addressed:

1. The study has provided a significant relationship on ABCG2 polymorphism gene in the risk of hyperuricemia on specific age of 18-25 years old. Detailed explanation has been provided for the reasons of relationship between ABCG2 polymorphism on hyperuricemia. However, are there any specific relations on the ABCG2 on specific young adults of age? Why perform the study in specific young adult age groups? Is it better to examine the relationship in a more general group?
2. Is there any consideration of excluding preexisting disease such as cardiovascular, liver, kidney, and secondary hyperuricemia (leukemia, myeloma, drugs induced, hypothyroidism, etc) in this study?
3. Is there any specific reason to choose the rs2231142 SNPs from all polymorphisms? The study should also describe the reasons to choose this rs2231142 SNP.
4. It has been revealed in the study that gender, obesity/overweight, hypercholesterolemia, and hypertriglyceridemia are associated with the risk of developing hyperuricemia. Therefore, in the analysis of the relationship of ABCG2 gene polymorphism and hyperuricemia, it is better to perform the analysis of other subgroups other than gender (such as BMI, cholesterol levels, and triglyceride)
5. The author should earlier define the diagnostic criteria used for hyperuricemia and its cut off value in the methods section.

Is the work clearly and accurately presented and does it cite the current literature?

Yes

If applicable, is the statistical analysis and its interpretation appropriate?

Yes

Are all the source data underlying the results available to ensure full reproducibility?

Yes

Is the study design appropriate and is the work technically sound?

Yes

Are the conclusions drawn adequately supported by the results?

Yes

Are sufficient details of methods and analysis provided to allow replication by others?

Yes

Reviewer Expertise:

Metabolic syndrome, hyperuricemia, insulin resistance, endocrinology, internal medicine, urology

I confirm that I have read this submission and believe that I have an appropriate level of expertise to confirm that it is of an acceptable scientific standard.

# Response

**Published:** 2021-04-02

**Authors:** Jorge Alegría

**Affiliations:** Universidad de Guanajuato, Mexico

Regarding the five points discussed, we respond to each of them:

 1. Although examining the relationship in a more general group could provide more information, this study included college applicants due to the facilities to be recruited. In addition, we directed the study to recognize risk factors in young population, an age group less likely to have metabolic diseases.

 2. In fact, participants with pre-existing diseases were not excluded. As mentioned in the methods section: “All participants were approached to take part in the study by phone and email by means of an open invitation explaining the goals of the study”.

 3. The ABCG2 (rs2231142) polymorphism is one of the most studied and it seems to have the greatest influence on uric acid levels in both men and women (Guo *et al*., 2020; Narang *et al*., 2019; Roman *et al*., 2020). Likewise, this polymorphism could be influencing both the mRNA expression levels and activity of ABCG2 modulated by cholesterol levels (Rodrigues *et al*., 2009; To *et al.,* 2014), as this was mentioned in the discussion. In relation to the first point, most studies of polymorphisms focus on older patients or hemodialysis patients; however, we studied young people.

 4. In order to summarize and present the most relevant results, the data were shown taking into account the genotype as a grouping criterion (Table 3). When the data was grouped and analyzed by another criterion (data not shown), there were no relevant findings.

 5. Serum uric acid ˃6 and 7 mg/dL was considered as hyperuricemia for women and men, respectively (Table 1). We considered it more appropriate to include this information where the study group data are shown.
